# O39b. Cascades antibacterial intelligent hand paper

**DOI:** 10.1186/1753-6561-5-S6-O90

**Published:** 2011-06-29

**Authors:** N Comeau

**Affiliations:** 1 Innovation Produits Hors Foyer-Amèrique Du Nord, Canada

## Introduction / objectives

A US study published in the Journal of Food Protection in 2006 shows that only 32% of food processing-industry workers wash their hands properly. Compliance with proper hand hygiene practices is less than 40% in the healthcare sector. After more than five years of research and development, Cascades entered the Intelligent paper™ market with a novel approach in the fight against the spread of bacteria by hand contact.

## Methods

Cascades took a commodity product like paper hand towel and transform it into a tool to prevent infection transmission by hands. This recent innovation by Cascades Tissue Group contributes to optimizing hand hygiene by combining effective drying with persistent antibacterial protection. The Intelligent™ antibacterial paper hand towel quickly reduces the amount of bacteria left on the hands after washing and provides antibacterial protection for 30 minutes afterward. The paper is referred to as Intelligent™ because it compensates for people’s imperfect hygienic habits without changing the way they do things. When drying your hands with the antibacterial hand towel, the water on your hands solubilises and releases benzalchonium chloride and transfers it onto the hands, thus reducing residual bacteria almost instantly.

## Results

Most importantly, it provides antibacterial protection for 30 minutes, reducing the risks of future contamination. The first application of this new market niche is an antibacterial hand towel with persistent action, which already has a provisional patent. Cascades antibacterial paper is a world premier that responds to unmet market needs.

## Conclusion

This domestic hygiene product reduces residual bacteria and compensates for people’s imperfect hygienic habits without changing the way they wash and dry their hands. It’s proven to be safe, effective and environmentally responsible.

## Disclosure of interest

None declared.

## Note

This abstract was presented as O39b.

